# Clinically-accessible and laboratory-derived predictors of biomechanical response to standalone and supported lateral wedge insoles in people with knee osteoarthritis

**DOI:** 10.1186/s13047-023-00671-7

**Published:** 2023-10-26

**Authors:** Michael A. Hunt, Calvin T. F. Tse, Michael B. Ryan, Alexander Scott, Eric C. Sayre

**Affiliations:** 1 Department of Physical Therapy, University of British Columbia, 212 – 2177 Wesbrook Mall, Vancouver, BC V6T 1Z3 Canada; 2 Motion Analysis and Biofeedback Laboratory, University of British Columbia, Vancouver, BC Canada; 3 Graduate Programs in Rehabilitation Sciences, University of British Columbia, Vancouver, BC Canada; 4 Kintec Footlabs Inc., Surrey, BC Canada; 5 School of Mechatronic Systems Engineering, Simon Fraser University, Burnaby, BC Canada; 6 British Columbia Centre On Substance Use, Vancouver, BC Canada

**Keywords:** Lateral wedge insoles, Knee, Osteoarthritis, Walking, Responder, Prediction, Logistic regression

## Abstract

**Background:**

Lateral wedge insoles (both standalone and those incorporating individualized arch support) have been frequently studied for the effects on knee joint loading and pain in people with knee osteoarthritis. It has been shown that many people who use these insoles do not obtain the intended biomechanical effect, and thus may not experience a clinical benefit. The ability to identify biomechanical responders to lateral wedge insoles before research or clinical intervention is an important objective for efficient resource use and optimizing patient outcomes. The purpose of our exploratory, hypothesis-generating study was to provide an initial assessment of variables that are associated with the biomechanical response to lateral wedge insoles in people with knee osteoarthritis.

**Methods:**

We collected a number of demographic (age, sex, body mass index, foot posture), clinical (knee pain, foot pain, radiographic disease severity), and walking-related (speed, knee alignment, frontal plane subtalar movement, and foot rotation) outcomes from 53 individuals with painful, radiographically-confirmed knee osteoarthritis. The walking-related outcomes were obtained using equipment both from the research laboratory and the clinical setting. We used logistic regression to generate predictive models to determine candidate variables associated with a reduction in the knee adduction moment during walking – a surrogate for tibiofemoral load distribution, and a known biomechanical risk factor for osteoarthritis progression – with the use of standalone and arch-supported lateral wedge insoles. Three different response thresholds (2%, 6%, and 10% reductions in the knee adduction moment) were used.

**Results:**

In general, biomechanical responders were those who walked faster, were female, had less varus alignment, and had less severe radiographic severity. Findings were similar between the standalone and arch-supported lateral wedge insoles, as well as between models using the laboratory-derived or clinically-available measures of walking performance.

**Conclusions:**

Our hypothesis-generating study provides valuable information that will inform future research into the efficient and effective use of lateral wedge insoles in the conservative management of knee osteoarthritis.

**Supplementary Information:**

The online version contains supplementary material available at 10.1186/s13047-023-00671-7.

## Background

Osteoarthritis (OA) of the knee is one of the most common musculoskeletal disorders, affecting more than 364 million individuals worldwide in 2019, resulting in an estimated 11.5 million years lived with disability globally [1]. There are more than 29 million incident cases annually [1], with the number of individuals affected, as well as the personal and economic burden, of knee OA expected to rise dramatically in the coming decades. In the absence of a cure, effective treatment strategies that are accessible and inexpensive are needed.

Lateral wedge insoles (LWIs) are a non-surgical, inexpensive, passive treatment approach for knee OA that has received much attention in the research literature. LWIs are designed to promote a lateralization of force distribution in the tibiofemoral joint, thereby offloading the more commonly affected medial compartment. This is achieved through a reduction in the external knee adduction moment (KAM) – a proxy for load distribution in the tibiofemoral joint during walking [2, 3], and a biomechanical risk factor obtained during instrumented motion analysis that has consistently been shown to be associated with the progression of structural changes associated with knee OA [4]. Systematic reviews have shown that, on average, the use of LWIs decrease the KAM by approximately 6% on every step taken [5, 6].

Despite the known biomechanical effect of LWIs, symptomatic improvement with their use is less clear based on findings from well-controlled randomized clinical trials – specifically, while significant improvements in pain and function have been shown with LWI use, the magnitude of these changes are not greater than using a neutrally-aligned control insole [7]. This lack of established superiority has resulted in clinical guidelines not recommending the use of LWIs in the clinical management of knee OA [8, 9]. Despite some evidence supporting an association between the cross-sectional magnitudes of KAM and knee pain [10, 11], as well as associations in changes in between these outcomes after an intervention [12, 13], a major limitation of the clinical trials used to inform these guidelines is the likely inclusion of biomechanical non-responders to LWIs, who would subsequently be less likely to improve pain under a biomechanically-driven framework.

Current evidence suggests that up to approximately one in three individuals with medial tibiofemoral osteoarthritis (TFOA) may be biomechanical non-responders with LWI use [14–17]. Specifically, rather than experiencing a reduction in the KAM with LWIs, non-responders demonstrate no change, or even an increase, in the KAM. If KAM reduction with LWI could be better identified through screening for biomechanical responses ahead of intervention, improvements in clinical outcomes such as pain and function may be achieved more reliably. Indeed, Felson et al. conducted a randomized controlled cross-over trial that pre-screened individuals with medial TFOA for a minimum 2% reduction in the early stance KAM peak with 5° LWIs [17]. From this cohort of biomechanical responders, it was demonstrated that the improvement in knee pain after intervention with LWIs for 8 weeks was superior to the change in pain after intervention with a neutral insole. Other attempts at identifying the KAM response prior to LWI intervention have shown promise biomechanically [18, 19] or clinically [20]. From both a clinical and research perspective, being able to identify people who are likely to be responders to treatment prior to an intervention is an important objective.

A primary limitation of previous attempts at identifying biomechanical response with LWIs use has been the reliance on motion capture and force platform technology. Although these tools provide precise and reliable measurements that would assist researchers to identify responders, they are generally exclusive to research settings and thus cannot be readily accessed by healthcare professionals that provide clinical care for individuals with knee OA. Therefore, developing clinically-accessible methods to predict biomechanical response to LWIs is necessary to improve the clinical application of this research, and ultimately the use of these insoles for managing medial TFOA. We are unaware of any studies that have used non-laboratory-based methods to predict biomechanical response to LWIs use.

Therefore, the objective of this exploratory, hypothesis-generating study was to assist in the development of prediction methods for identifying biomechanical responders to LWIs (both standalone and those incorporating person-specific arch support) using clinically-accessible measurements of demographics, anthropometrics, descriptors of knee OA, as well as joint alignment and motion as predictor variables. Given the similar relevance to researchers, we also conducted analyses using traditional laboratory-based outcomes.

## Methods

### Participants

Individuals with knee OA were recruited from the community using paper-based and online advertisements between October 2018 and October 2021 (including a required research shutdown due to the COVID-19 pandemic). Eligible participants had to be 50 years of age or above and have radiographically confirmed OA predominantly in the medial tibiofemoral compartment assessed using the Kellgren and Lawrence (KL) classification scale [21] from coronal plane radiographs of the tibiofemoral joint obtained during upright standing. Participants also had to demonstrate the following inclusion criteria: greater joint space narrowing in the medial compartment than lateral compartment; a history of knee pain longer than six months; and a minimum average knee pain of 3 out of 10 (0 = “no pain”; 10 = “worst pain imaginable”) in the 1-month period preceding study participation. Any individual with any history of the following were excluded from study participation: lower-limb surgery or joint injections in the preceding 6-months; any injury or dysfunction that impaired standing balance or walking ability in the 12-months preceding study participation; and consistent use of orthotic insoles in the 12-months preceding study participation. All participants provided written informed consent, and the study was approved by the institution’s research ethics board for clinical studies.

### Insoles

Eligible participants were referred to a Certified Canadian Pedorthist for final confirmation of study eligibility and to undergo 3D laser volumetric casting of their feet, taken in a non-weightbearing subtalar neutral position. Three pairs of sulcus length orthotic insoles were custom-fabricated for each participant, and finished with an identical neoprene cover. Neutral 3 mm flat control (FLAT) and 5° lateral wedge (WEDG) insoles were made from ethyl-vinyl acetate foam (EVA) (Shore A hardness = 55). One pair of variable stiffness custom contoured arch supports incorporating a lateral wedge (WEDG + V-ARCH) were formed from the volumetric casts using plastazote foam laterally (Shore A hardness 70) and EVA medially (Shore A hardness 20). Every pair of insoles was sent directly to the University; upon receipt, participants were invited to the laboratory for a single testing session.

### Data collection

The index side was defined as the one with knee pain and radiographic evidence of osteoarthritis in the case of unilateral symptoms, or the more painful knee in cases of bilateral symptoms and radiographic evidence. Participants first completed questionnaires for the index limb, including a numerical rating scale of average knee pain over the previous week (numerical rating scale (NRS) pain: 0 = “no pain”; 10 = “worst pain imaginable”), as well as the Foot Function Index (FFI) questionnaire (revised, short form) [22]. For the purposes of this study, we only used the foot pain subscale (FFI pain), and all scores were converted to a percentage score (25% = least pain; 100% = most pain), as per guidelines. A number of anthropometric measurements were taken, including height and body mass.

**Fig. 1:**
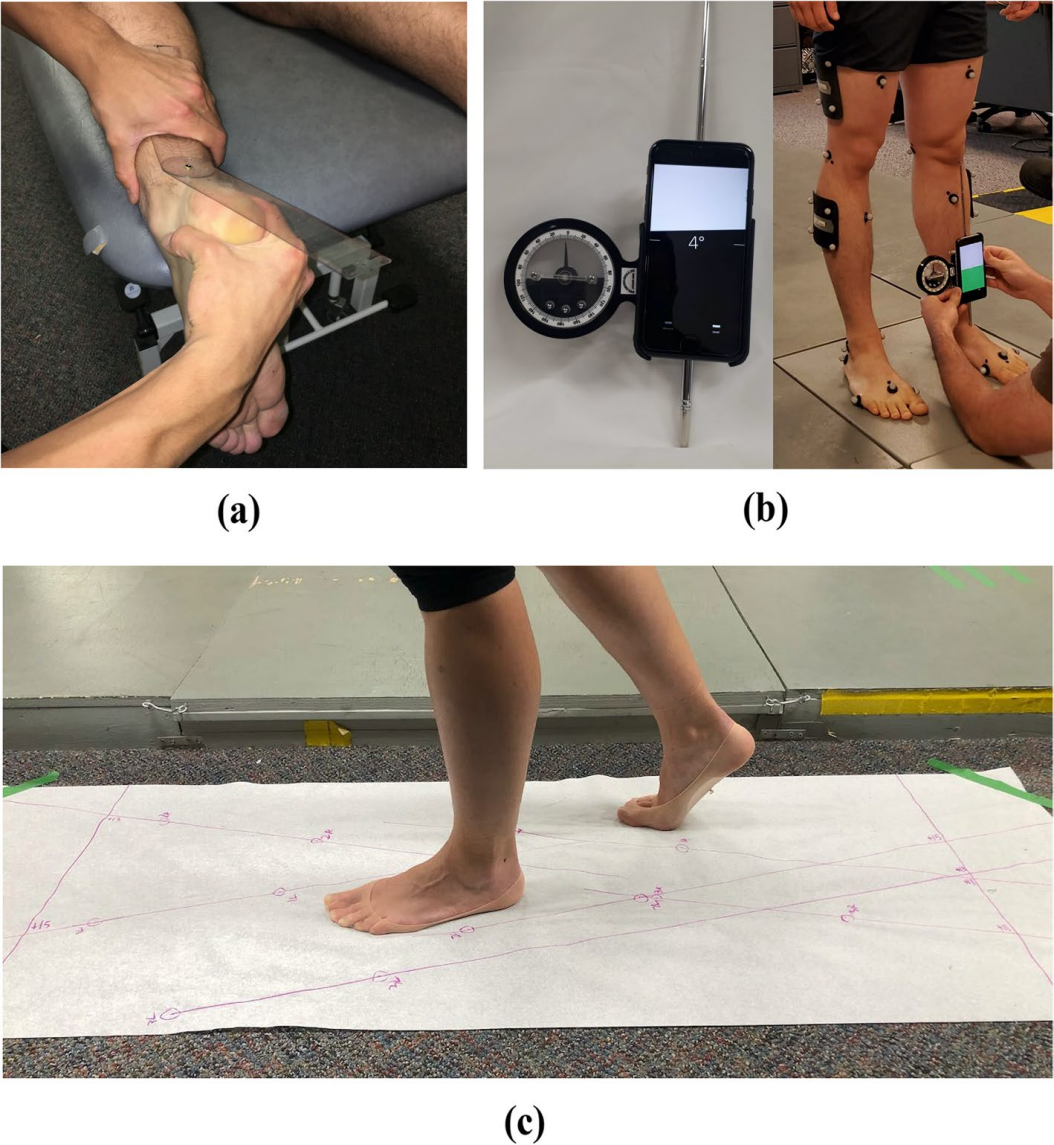
Visualizations of the collection of the data using clinically-accessible methods: **a** Ankle/subtalar eversion range of motion was measured using goniometry; **b** Frontal plane tibial angle was measured using a smartphone inclinometer with an external alignment device collinear to the long axis of the smartphone (left). The participant position during measurement of frontal plane tibial angle is shown on the right; **c** Foot progression angle was measured using a wet sock as the participant walked across a length of exam paper

We also measured a number of outcomes shown to be correlated with KAM magnitudes (see Fig. 1):

i) The six-item Foot Posture Index (FPI) assessment was conducted by a trained assessor to provide a numerical rating of the foot posture of the index foot for each participant [23]. The numerical sum of the six FPI items for the for each participant was used for this study.
ii) Passive subtalar joint eversion range of motion of the index foot was measured with manual goniometry, which has previously demonstrated acceptable relative intra-rater measurement reliability [24]. Measurements were taken with participants resting in a prone position with the foot and ankle hanging off the end of a plinth, where the calcaneus was passively moved by the assessor until a firm end feel was detected. Peak range was recorded, and the average of three measurements was calculated.
iii) Frontal plane tibial angle of the index limb was measured during relaxed standing via smartphone inclinometry supplemented with an external alignment device. This technique demonstrates adequate measurement validity compared to motion capture, as well as excellent inter-rater and inter-session measurement reliability [25]. Participants were barefoot and stood with knees extended, feet facing forward, and weight comfortably distributed between both legs. The alignment device was aligned with the centre of the tibial tuberosity and neck of the talus before reading the smartphone inclinometer value. Participants briefly marched on the spot between each measurement to reset their standing position, and the mean of three measurements was calculated.
iv) Gait speed was measured using photoelectric timing gates positioned 4 m apart in the middle of a 10 m walkway. Participants completed three passes along the full length of the walkway, and the mean speed across all trials was calculated.
v) Foot progression angle was measured as participants walked across a 3 m length of medical exam paper while donning wet nylon socks that left an imprint of the paper. Foot progression angle was measured as the angle between the foot axis (heel centroid to tip of 2^nd^ toe) and a line corresponding to walking direction. The mean of three trials was calculated.

Participants then underwent 3D gait analysis using motion capture technology. Forty-seven retroreflective markers were affixed to the skin over anatomical landmarks on the pelvis and lower body, including: the sacrum, and bilaterally over the anterior and posterior superior iliac spines, lateral femoral epicondyles, lateral malleoli, posterior aspects of the calcanei, and heads of the second metatarsals. Tracking markers were placed bilaterally on the lateral thighs and shanks as rigid plates (four markers each), bilaterally on the anterior thighs and shanks, as well as on either side of the posterior calcanei markers forming a heel triad. Ten of the forty-seven markers were affixed bilaterally only during a static calibration trial, including: greater trochanters, medial femoral epicondyles, medial malleoli, and first and fifth metatarsal heads.

Participants completed walking trials across a 10 m walkway, with kinematic data collected at 100 Hz from 14 cameras (Motion Analysis Corp., Santa Rosa, CA) synchronized with two floor-embedded force platforms (AMTI Inc., Watertown, MA) sampling at 2000 Hz. All gait testing always occurred with the FLAT condition first, with the order of the other two conditions (WEDG or WEDG + V-ARCH) systematically randomized following a Williams design (2 × 2; AB BA) to minimize any condition ordering effect. The mean of five successful walking trials represented the laboratory-derived gait data for each insole condition. A walking trial was deemed successful only when index foot completed the stance phase entirely within the boundaries of the force platform, and the walking speed was maintained within ± 5% of the self-selected walking speed established during walking trials with FLAT.

### Data processing

Using a previously-published biomechanical model [26] inverse dynamics calculations combined segmental kinematic and force data to calculate 3D joint forces and moments using commercially available software (Visual 3D; C-motion, Rockville, MD). The KAM impulse (Nm/kg*sec) was selected as the outcome of interest for defining biomechanical response as it is representative of the cumulative loading across the duration of the stance phase of gait and can be obtained with acceptable test–retest measurement reliability [27]. We also identified the following 3D gait values from the FLAT trials to serve as predictor variables: rearfoot excursion (°, frontal plane range between initial contact and the time of peak eversion), frontal plane knee angle (°, mean value between 25 and 50% of stance), gait speed (m/sec), and foot progression angle (°, mean value between 15 and 50% of stance).

### Statistical analysis

The primary objective of the study was to identify biomechanical responders to LWI use, and this was achieved using logistic regression. The dependent variable used in all logistic regression modelling was the binary biomechanical responder classification and for each LWI condition (WEDG and WEDG + V-ARCH) separately. We created regression models for a variety of KAM impulse response thresholds (decreases of 2%, 6%, or 10% compared to the FLAT condition) using both Akaike Information Criterion (AIC) and forced-entry modelling (see Supplementary File 1 for description). The 2% threshold corresponds with the threshold used by Felson et al. [17] shown to be associated with a greater likelihood of pain improvement, while the 6% threshold approximates the average KAM reduction shown in meta-analyses [5, 6]. The additional 10% threshold was chosen as an incremental increase from 2 and 6%, such that anyone predicted to be a responder at this threshold would be likely to experience a greater reduction in the KAM with LWIs.

Two separate model lists were created that included demographic, anthropometric, and disease-related outcome predictor variables and outcomes derived either using clinically-accessible methods or laboratory-derived 3D motion capture technology. A summary of these models can be found in Table 1.

**Table 1:** Predictor variables separated by category of participant information. Note that 2 separate groups of models were used: Demographic, anthropometric, and knee OA descriptor variables were used in all models, while the gait, posture, and movement characteristics were separated by whether they were derived using clinically accessible or laboratory-derived approaches. Note that the laboratory-derived data were from the FLAT condition trials

**Demographics, anthropometrics, and descriptors of knee osteoarthritis**		
Age (years)		
Sex (male/female)		
Body mass index (kg/m^2^)		
Foot posture index (-12 to + 12)		
Numerical rating scale of knee pain (0 to 10)		
Foot function index pain score (normalized 25% to 100%)		
Kellgren & Lawrence grade (dichotomized as KL2 or KL3–4)		
**Gait, posture, and movement characteristics**		
	*Clinically-Accessible*	*Laboratory-Derived*
Gait speed (dm/s)	Fixed distance between two photoelectric timing gates, divided by time elapsed to cross from one gate to the other	The distance between heel strike to heel strike, divided by time elapsed between the heel strike events
Ankle/subtalar eversion motion ( ° )	Range between resting posture and end range eversion	Frontal plane ankle/subtalar joint angle excursion between initial contact and peak eversion
Foot progression angle ( ° )	Foot position measured during walking over a length of paper with wet socks	Mean foot progression angle during midstance (15 to 50% normalized stance)
Frontal plane knee alignment ( ° )	Tibial inclination in frontal plane, measured by digital smartphone inclinometer	Mean frontal plane knee angle during midstance (25 to 50% normalized stance)

In the initial step of the analysis, predictor variables were checked for collinearity with Spearman rank correlations. Since a 1.0 m/s unit change in gait speed is larger than can be meaningfully interpreted as a predictor variable, clinically-accessible and laboratory-derived gait speed values were multiplied by 10 for use in the prediction models. By doing so, the scale of interpreting gait speed from prediction model outputs was improved, such that a single unit difference in gait speed represented a change of 1.0 dm/s (10 cm/s), rather than 1.0 m/s. Univariable logistic regression models were fit and the odds ratios for each predictor variable were explored for the directionality and strength of relationship between the predictor variable and biomechanical response.

Multivariable logistic regression models were fit using two levels of variable selection. At the first level, a forward selection and backwards elimination stepwise process selected a set of possible predictor variables from each pool of clinically-accessible or laboratory-derived predictor variable inputs at α = 0.30. This level of variable pre-selection eliminated any predictor variable that was unlikely to remain as a significant predictor in the final model, and also determined the order of predictor variable entry for the next level of variable selection. The second level of variable selection used the AIC approach to determine the final set of variables in each model. Predictor variables were entered into the logistic model in a stepwise fashion until the addition of a predictor variable increased the AIC from the previous step. Only predictor variables that were entered into the model before the AIC increased were included in the final prediction model.

The omnibus effect of each model was determined to be significant if the model likelihood ratio was significant and the Hosmer and Lemeshow goodness of fit test statistic was non-significant at *p* > 0.05. The predictive utility of each model was assessed via the area under the curve (AUC) of the receiver operating characteristic (ROC) curve, c, along with its 95% confidence limits. The odds ratio for each predictor variable indicated the factor by which the odds of being classified as a biomechanical responder changed per unit increase in the predictor variable. An odds ratio > 1.0 represented a greater odds of a biomechanical responder classification, and < 1.0 represented a lower odds of biomechanical responder classification in each particular combination of response threshold and LWI condition. A predictor variable was considered significant in the final AIC-selected model if the *p*-value for its odds ratio was *p* < 0.05. Predictor variables with an odds ratio that had a *p*-value 0.05 < *p* < 0.10 were considered predictor variables of interest. All analyses were performed using the statistical software SAS v9.4 (SAS Institute, Cary, NC).

## Results 

Fifty-three individuals with medial TFOA (39 females; mean (SD) age = 64.4 (6.9 years); mean (SD) BMI = 26.6 (3.9) kg/m^2^) participated in this study. A summary of descriptive statistics of the demographic, anthropometric, descriptors of knee OA, as well as gait, posture, and movement predictor variables are found in Table 2. Descriptive differences in discrete outcomes of knee and ankle joint angles and moments between insole conditions are presented in Supplementary File 2.

**Table 2:** Descriptive statistics for predictor variables. Most values reported as mean ± standard deviation (25^th^, 75^th^ percentile), with exceptions noted below

**Anthropometrics, and descriptors of knee osteoarthritis**		
Foot posture index (-12 to + 12)	5 (2,8)	
Numerical rating scale knee pain (0 to 10)	4.4 ± 2.1 (3.0, 6.0)	
Foot Function Index pain score (25% to 100%)	43.3 ± 17.0 (30.0, 50.0)	
Kellgren and Lawrence grade (KL2—KL3—KL4)	27 – 22 – 4	
**Gait, posture, and movement characteristics**		
	Clinically-Accessible	Laboratory-Derived
Gait speed (dm/s)	11.9 ± 1.8 (10.9, 13.2)	11.8 ± 1.8 (10.7, 12.9)
Ankle/Subtalar eversion motion ( °)	8.8 ± 3.9 (6.0, 11.3)	7.0 ± 2.7 (5.6, 8.2)
Foot progression angle ( °)^a^	-9.5 ± 6.0 (-12.0, -6.0)	-10.9 ± 6.8 (-13.6, -6.3)
Frontal plane tibial/knee angle ( °)^a^	-1.1 ± 3.5 (-3.3, 0.7)	-2.0 ± 5.0 (-4.6, 1.9)

Foot posture index reported as median (25^th^, 75^th^ percentile)

KL grade reported as number of observations

^a^negative values represent toe-out foot progression angle, or knee varus alignment angle

From the exploratory univariable logistic regression models, several predictor variables demonstrated significant models for predicting the biomechanical response to WEDG and WEDG + V-ARCH at multiple response thresholds (Supplementary File 3). In general, a greater likelihood of KAM impulse reduction with both insoles was associated with faster walking speeds, less varus aligned knees, and a lower radiographic severity of medial TFOA.

To succinctly display the results from multivariable regression analyses, only model statistics and odds ratios for the 2% response threshold are summarized in Table 3. The model statistics and odds ratios for multivariable logistic models for the 6% and 10% response thresholds selected via the AIC approach are summarized in Supplementary File 4, while all model data (2%, 6%, and 10%) using the forced-entry approach are summarized in Supplementary File 5. In all situations, only models that converged statistically and achieved a significant omnibus effect are reported.

**Table 3:** AIC-selected logistic regression model statistics for 2% response threshold. Bolded odds ratio values and *p*-values indicate statistical significance (α = 0.05)

		**WEDG**		**WEDG + V-ARCH**	
		**Clinically-Accessible**	**Laboratory Derived**	**Clinically-Accessible**	**Laboratory Derived**
Responder: Non-Responder		33:20	33:20	28:25	28:25
Model AIC		58.975	59.445	65.252	63.453
Model Likelihood Ratio (p-value)	*p*	**< *0.001***	**< *0.001***	***0.001***	**< *0.001***
H&L Goodness of Fit (p-value)	*p*	0.815	0.575	0.074	0.109
AUC ROC(c)	c	0.859	0.794	0.829	0.843
	95%CI	(0.753, 0.965)	(0.670, 0.918)	(0.709, 0.949)	(0.729, 0.957)
		**Odds Ratios by Predictor Variable**			
		**Gait speed (Clin)**	**Gait speed (Lab)**	**Gait speed (Clin)**	**Gait speed (Lab)**
	OR	**3.118**	**2.224**	**2.040**	**2.240**
	95%CI	**(1.465, 6.633)**	**(1.301, 3.795)**	**(1.243, 3.349)**	**(1.315, 3.815)**
	*p*	***0.003***	***0.003***	***0.005***	***0.003***
		KL Grade	KL Grade	**Sex**	**Sex**
	OR	0.380	0.374	**0.180**	**0.157**
	95%CI	(0.090, 1.602)	(0.099, 1.416)	**(0.034, 0.954)**	**(0.028, 0.888)**
	*p*	0.188	0.148	***0.044***	***0.036***
		**FFI Pain**		**FFI Pain**	**FFI Pain**
	OR	**0.948**		**0.948**	**0.948**
	95%CI	**(0.900, 0.999)**		**(0.904, 0.995)**	**(0.903, 0.994)**
	*p*	***0.046***		***0.029***	***0.029***
		NRS Pain		NRS Pain	NRS Pain
	OR	1.609		1.463	1.512
	95%CI	(0.996, 2.598)		(0.974, 2.199)	(0.991, 2.308)
	*p*	0.052		0.067	0.055
		FPA (Clin)			
	OR	0.901			
	95%CI	(0.779, 1.043)			
	*p*	0.164			

*Abbreviations*: *AIC* Akaike information criterion, *AUC* Area under curve, *BMI*  body mass index, *FFI* foot function index, *FPI* foot posture index, *H&L* Hosmer & Lemeshow, *KL* Kellgren & Lawrence, *OR* odds ratio, *ROC* receiver operating characteristic

For the WEDG condition at the 2% response threshold, clinically-accessible and laboratory-derived gait speed was a significant predictor of biomechanical response, such that a faster gait speed was related to an increased likelihood of reducing the KAM impulse. Additionally, FFI pain was a significant predictor in the clinically-accessible model only, such that a lower level of foot-related pain was related to a greater likelihood of reducing the KAM impulse with WEDG. Only gait speed was a significant predictor of response with WEDG at 6%, while only KL grade significantly predicted response with WEDG at 10%.

For the WEDG + V-ARCH condition at the 2% response threshold, the models using clinically-accessible and laboratory-derived predictor variable inputs shared similar significant predictors variables selected by AIC. Gait speed (faster speed), and FFI pain (less foot pain) were significant predictors in the models, as was female sex. Similarly, faster gait speeds, female sex, and lower KL grades were significant predictors in models for WEDG + V-ARCH at 6%, while faster gait speeds, less varus knee alignment, and older age were significant at the 10% KAM threshold.

Across the 12 different combinations of AIC-selected models (three response thresholds (2%, 6%, 10%) × two pools of predictor variable inputs (clinically-accessible, laboratory-dervied) x two LWI conditions (WEDG, WEDG + V-ARCH)), the number of occurrences that a predictor was significantly selected may be an indicator of its importance for predicting the KAM impulse response with LWIs. The number of occurrences a predictor variable was significantly selected in the final models (*p* < 0.05) included: gait speed (9/12), sex (4/12), FFI pain (3/12), knee angle (2/12), KL grade (2/12), and age (1/12). Additionally, the number of occurrences for predictor variables of interest (0.05 < *p* < 0.10) from the 12 possible AIC-selected models included: KL grade (4/12), knee angle (3/12), NRS pain (3/12), age (2/12), sex (2/12), and gait speed (1/12). Predictor variables that were not selected as a significant predictor or predictor variable of interest in any AIC-selected models included BMI, FPI, and both clinically-accessible and laboratory-derived versions of FPA and ankle/subtalar eversion motion.

## Discussion 

The current hypothesis-generating study was designed to develop prediction models for identifying a reduction in the KAM impulse during gait with standalone or supported-LWI in individuals with medial TFOA. We have provided for the first time, to our knowledge, data on the predictive capacity of a variety of variables available in the clinical setting that can be used to determine the potential biomechanical response to LWIs. Given the hypothesis-generating nature of this study, we provided data based on different biomechanical response thresholds and different regression modelling approaches so that researchers and clinicians can determine the best use of our findings based on their unique resources, expected response, and needs. Faster gait speed emerged most frequently as a significant predictor variable, with additional significant predictor variables including female sex, and lower FFI pain. While not statistically significant at *p* < 0.05, other variables such as lower KL grade, higher NRS pain, and less varus alignment emerged as variables of interest at the 0.05 < *p* < 0.10 level of significance. By providing a comprehensive report on response likelihood across different KAM reduction thresholds, different insole conditions, and different methods of acquiring predictor data, we feel that information from this current work may be able to guide researchers and clinicians in their consideration for LWI prescription for medial TFOA management, and to inform future research regarding variables that may be worth investigation in refinements of our models to predict LWI response biomechanically.

Individual predictor variables demonstrated varying abilities to distinguish between biomechanical responders and non-responders to LWIs. We found self-selected gait speed, regardless of its clinically-accessible or laboratory-derived origin, was the most influential in predicting a KAM impulse reduction, with significantly greater odds of experiencing a reduction in the KAM impulse with LWIs for every unit increase (1 dm/s) in gait speed across 9/12 AIC-selected models. Faster gait speeds are known to be associated with a larger magnitude of KAM peak [28, 29]. Since gait speed was constrained between insole conditions, higher baseline magnitudes of KAM – from a faster self-selected gait speed – may potentially provide more opportunity for an LWI to impart a KAM-reducing effect. Our modelling suggests that the odds of reducing the KAM impulse with LWIs is greater in individuals who are female, older, and have less foot-related pain, less varus knee alignment, and a lower KL grade. While we did not investigate the mechanisms for KAM reduction with LWIs related to each of these predictor variables, this list may highlight variables that should be prioritized to obtain in clinical settings for predicting biomechanical response when resources are limited or that are worth future investigation in prospective biomechanical prediction models.

The predictive ability of clinically-accessible and laboratory-derived prediction models appeared to perform similarly, which suggests more resource intensive methods for obtaining relevant data may not always be necessary. Since values of the AUC of ROC curve were not statistically compared, we could not formally validate clinically-accessible prediction models against their laboratory-derived counterpart. However, using the AIC-selected model at the 2% response threshold for WEDG + V-ARCH as an example, the c [95%CI] values for the clinically-accessible (c = 0.829 [0.709, 0.949]) and laboratory-derived (c = 0.843 [0.729, 0.957]) appeared to be similar. Furthermore, the significant predictor variables selected by AIC for a given response threshold and insole condition were generally similar between clinically-accessible and laboratory-derived models; this provides confidence that the clinically-accessible predictors are appropriately representing the same constructs as their laboratory-derived counterparts. A similar trend was found for clinically-accessible and laboratory-derived models matched for the same combination of response threshold and LWI condition.

While speculative, our current findings may shed light on previous LWI clinical research. Our findings present a picture that those with knee OA who are more likely to respond biomechanically to LWIs are less impacted by the disease – those that are more functional (ie. walk faster), have less varus malalignment, and have lower radiographic severity. Given that the likely mechanical effect of a 5 degree LWI is relatively low, it stands to reason that those with more varus malalignment and structural degradation would require a larger mechanical intervention to produce the biomechanical realignment that would reduce the KAM. These findings may translate to improvements in symptoms as well. Indeed, when assessing pain improvement with the use of LWIs, Baker et al. showed in their sub-group analysis that individuals with KL < 4 improved their pain by 21 points (out of 500) on the Western Ontario and McMaster Universities Osteoarthritis Index, while those with KL = 4 improved by only 2 points [30] following six weeks of use. Clinical trials may be best operationalized by specifically recruiting those with earlier stage disease, though future research is needed to examine phenotypes of those who symptomatically respond to LWIs.

Predictor variables that were not selected into any AIC-selected models suggests that these metrics may not be useful for predicting biomechanical response to LWIs in the presence of all other chosen predictor variables. Static foot posture, represented by FPI, did not significantly contribute to AIC-selected models as a predictor. This finding aligns with a report that metrics of static foot structure, excluding FPI, cannot predict biomechanical responses to wedged footwear in individuals with medial TFOA [18]. Ankle/subtalar eversion motion had previously been shown to predict biomechanical responders from non-responders to LWI treatment [14]. However, the influence of frontal plane knee alignment on mediating the association between ankle/subtalar eversion and the KAM magnitude [31] may also be influencing the KAM response with LWIs observed in our study. Clinically-accessible and laboratory-derived frontal plane knee alignment was a significant predictor in two AIC-selected models, and was a predictor variable of interest in four others. Taken together, frontal plane knee alignment may prove to be more useful in predicting biomechanical response to LWI than ankle/subtalar eversion. Lastly, FPA was not a significant predictor in any model, which contrasts a previous report that larger reductions in the KAM with LWI occurred in healthy adults with a smaller natural FPA than those with a larger FPA [19]. Our findings regarding FPA may differ from previous works because our data were sampled from individuals with medial TFOA, and was also evaluated amongst the influence of a concert of predictor variables which may produce a different result than when FPA is studied in isolation.

The findings from this study should be interpreted with the following limitations in mind. Firstly, given the number of predictor variables that were explored for their predictive capabilities, a larger sample size may have been warranted to improve the confidence and generalizability of our prediction models. Particularly at higher response thresholds of KAM impulse reduction, the observed distribution of biomechanical responders to non-responders (e.g. 12 responders: 41 non-responders at 10% response threshold for WEDG + V-ARCH) was less proportional than the distribution at a lower threshold (e.g. 28 responders: 25 non-responders at 2% response threshold for WEDG + V-ARCH). Although our sample size (*n* = 53) was still larger than previous attempts at predicting biomechanical response with LWI, and the AIC method was selected to minimize the effect of model overfitting in light of this sample size, future studies would benefit from even larger sample sizes to obtain a greater spread of data to avoid model overfitting. Next, our selection of clinically-accessible measurements of gait, posture, and movement characteristics was driven by evidence from previous literature which suggested they could be relevant to KAM response with LWI. This is by no means an exhaustive list of possible biomechanical influences and other outcomes may emerge as relevant predictors in future research. However, since the clinically-accessible and laboratory-derived versions of predictor variables tended to be significant in the same iteration of response threshold and LWI condition, this gave us confidence that the clinically-accessible metrics adequately represented the same construct as their laboratory-derived counterparts. Finally, our clinically-accessible measure of gait speed was derived from photoelectric timing gates that may not be available in some clinical settings. While less accurate, gait speed is commonly measured clinically using a stopwatch and fixed distance marked on the floor that will likely provide an adequate assessment of gait speed for the purposes of identifying potential responders to LWIs. Future research to confirm the validity of clinically-accessible predictors as a surrogate for their corresponding laboratory-derived counterparts would strengthen the findings of the current study.

## Conclusion

The current investigation demonstrated the potential of predicting the KAM impulse response to LWIs using clinically-accessible and laboratory-derived predictor variables. Using predictor variables including gait speed, sex, knee and foot pain, it is feasible to predict a minimum 2% reduction in the KAM impulse with a standalone LWI and a supported-LWI in the clinical setting. Armed with biomechanical prediction tools, clinicians may be able to navigate their decision making around LWI prescription by predicting an individual’s likelihood of receiving a biomechanical benefit prior to insole intervention. Based on the results of our exploratory, hypothesis-generating study, future research can continue to refine prospective biomechanical prediction models by scrutinizing the predictor variables identified by this study to be of particular interest to the KAM impulse response with LWIs. By generating predictive tools to improve the precision of biomechanical intervention, the treatment efficacy LWIs may be improved for the conservative management of medial TFOA.

### Supplementary Information

**Additional file 1:** **Supplementary File 1.** Description of forced entry multiple regression models.

**Additional file 2:** **Supplementary File 1.** Knee and ankle biomechanical outcomes across insole conditions.

**Additional file 3: Supplementary File 3.** Univariable logistic regression model statistics.

**Additional file 4: Supplementary File 4a.** AIC-selected logistic regression model statistics for 6% response threshold. **Supplementary File 4b.** AIC-selected logistic regression model statistics for 10% response threshold.

**Additional file 5: Supplementary File 5.** Overview of findings using force-entry modelling.

## Data Availability

The datasets analyzed during the current study are available from the corresponding author on reasonable request.

## Abbreviations

AIC: Aikaike Information Criterion
AUC: Area under curve
EVA: Ethyl vinyl acetate
FFI: Foot Function Index
FLAT: 3 Mm EVA flat control insole
FPI: Foot Posture Index
KL: Kellgren and Lawrence radiographic classification
KAM: Knee adduction moment
LWI: Lateral wedge insole
NRS: Numerical rating scale
OA: Osteoarthritis
ROC: Receiver operating characteristic
TFOA: Tibiofemoral osteoarthritis
WEDG: 5° Lateral wedge insole
WEDG + V-ARCH: Lateral wedge with variable stiffness contoured arch support
