# Supplementary material for: Clinically-accessible and laboratory-derived predictors of biomechanical response to standalone and supported lateral wedge insoles in people with knee osteoarthritis
Source: J Foot Ankle Res. 2023 Oct 26;16:74. doi: 10.1186/s13047-023-00671-7 (PMC10601168; doi:10.1186/s13047-023-00671-7)
Supplement: Supplementary file 1 — Additional file 1: Supplementary File 1. Description of forced entry multiple regression models. [file 13047_2023_671_MOESM1_ESM.pdf]

Supplementary File 1 - Description of forced entry multiple regression models

In these models, all predictor variables from the set of clinically-accessible or laboratory-derived were force entered into the model to predict the binary biomechanical response at each combination of response threshold and LWI condition. With two sets of predictor variables (clinically-accessible or laboratory-derived), three response thresholds (2%, 6%, or 10%), and two LWI conditions (WEDG or WEDG+V-ARCH), a total of 12 forced-entry logistic regression models were calculated. Forced entry of all predictor variables from a given set of inputs may put a model at risk of overfitting. However, the odds ratio for each predictor variable and its predictive ability can be interpreted amid the adjusted effects of all other predictor variables. As such, the combined effects of multiple predictor variables can be explored for their possible contribution to predicting the binary biomechanical response. Specifically, a predictor variable was considered significant in the model if the p-value of its odds ratio was $p < 0.05$, and was flagged as a predictor variable of interest in the logistic model if the p-value of its odds ratio was $0.05 \leq p < 0.10$.

The omnibus effect of these models was deemed to be valid for interpretation if the likelihood ratio for the model was significant ($p < 0.05$), and the Hosmer and Lemeshow goodness of fit test statistic was non-significant ($p > 0.05$). A significant omnibus effect for a model indicated that the predictor variable inputs jointly had the capability to predict the binary biomechanical response to the corresponding LWI condition. The Hosmer and Lemeshow statistic was used to assess the goodness of fit between predicted and observed biomechanical responses; a significant test statistic indicated that the observed and predicted biomechanical responses were different

from one another, and thus the model is poorly fit. Additionally, receiver operating characteristic (ROC) curve analyses were performed to evaluate the predictive ability of each set of predictor variable inputs for classifying the binary biomechanical response at each combination of response threshold and LWI condition. The predictive ability of each model for correctly classifying the binary biomechanical response to LWIs was assessed by calculating the AUC of the ROC curve and its 95% CI, represented by the symbol, c. The c metric captures the joint specificity and sensitivity of the model as the threshold for positive prediction is varied, and values of c can lie between 0 and 1.0. A c value of 0.50 indicates that the model predicts the biomechanical response as well as pure random chance, and values closer to 1.0 indicate a model that has predictive ability superior to random chance. We have selected the following thresholds for general interpretation of c values: acceptable ($0.7 \leq c < 0.8$), good ($0.8 \leq c < 0.9$), excellent ($c \geq 0.9$) [24]. Note that while we cannot definitively draw conclusions about variables of interest, they may be considered good candidates for future research.
