# Supplementary material for: Clinically-accessible and laboratory-derived predictors of biomechanical response to standalone and supported lateral wedge insoles in people with knee osteoarthritis
Source: J Foot Ankle Res. 2023 Oct 26;16:74. doi: 10.1186/s13047-023-00671-7 (PMC10601168; doi:10.1186/s13047-023-00671-7)
Supplement: Supplementary file 2 — Additional file 2: Supplementary File 1. Knee and ankle biomechanical outcomes across insole conditions. [file 13047_2023_671_MOESM2_ESM.pdf]

Supplementary File 2. Knee and ankle biomechanical outcomes across insole conditions. Values are reported as mean ± standard deviation.

	Insole Condition		
**Outcome**	FLAT	WEDG	WEDG + V-ARCH
KAM peak (Nm/kg)	0.50 ± 0.14	0.49 ± 0.14 [a]	0.49 ± 0.14
KAM impulse (Nm/kg•sec)	0.18 ± 0.08	0.17 ± 0.08 [a]	0.18 ± 0.08
Knee frontal plane angle midstance (°) *	-2.0 ± 5.0	-2.0 ± 5.1	-2.1 ± 5.1
Ankle/subtalar eversion moment peak (Nm/kg) *	-0.14 ± 0.08	-0.17 ± 0.08 [a]	-0.15 ± 0.07 [b]
Ankle/subtalar eversion moment impulse (Nm/kg•sec) *	-0.04 ± 0.02	-0.06 ± 0.03 [a]	-0.05 ± 0.03 [a]
Ankle/subtalar eversion angle peak (°) *	-1.7 ± 3.8	-2.5 ± 3.8 [a]	-3.2 ± 4.1 [a,b]
Ankle/subtalar eversion excursion (°)	7.0 ± 2.7	7.2 ± 2.7	7.0 ± 2.5

[a] and [b] denote a significant difference from FLAT and WEDG, respectively ($p < 0.05$).

* Negative values denote knee varus and ankle/subtalar eversion.
