# Supplementary material for: Clinically-accessible and laboratory-derived predictors of biomechanical response to standalone and supported lateral wedge insoles in people with knee osteoarthritis
Source: J Foot Ankle Res. 2023 Oct 26;16:74. doi: 10.1186/s13047-023-00671-7 (PMC10601168; doi:10.1186/s13047-023-00671-7)
Supplement: Supplementary file 3 — Additional file 3: Supplementary File 3. Univariable logistic regression model statistics. [file 13047_2023_671_MOESM3_ESM.pdf]

Supplementary File 3. Univariable logistic regression model statistics. Data are presented for all predictor variables, response thresholds, and lateral wedge insole conditions for models that correspond to the clinically-accessible (Clin) and laboratory-derived (Lab) versions of the predictor variable, respectively. Bolded odds ratio values and p-values indicate statistical significance ($p < 0.05$). Unbolded odds ratios values and p-values indicate a predictor variable of interest ($0.05 < p < 0.10$). Odds ratios values and p-values were omitted if the predictor variable did not minimally reach the threshold of interest ($p \geq 0.10$).

		WEDG			WEDG+V-ARCH		
	Response Threshold	**2%**	**6%**	**10%**	**2%**	**6%**	**10%**
Age	OR<br>95%CI<br>*p*	-	-	-	-	-	-
Sex	OR<br>95%CI<br>*p*	-	-	-	-	0.333<br>(0.095,<br>1.173)<br>*0.087*	-
BMI	OR<br>95%CI<br>*p*	-	-	-	-	-	**0.802**<br>**(0.653,**<br>**0.985)**<br>***0.035***
FPI	OR<br>95%CI<br>*p*	-	-	-	-	-	-
NRS pain	OR<br>95%CI<br>*p*	-	-	-	-	-	-
FFI pain	OR<br>95%CI<br>*p*	0.971<br>(0.937,<br>1.005)<br>*0.096*	-	-	-	-	-
KL grade	OR<br>95%CI<br>*p*	0.350<br>(0.110,<br>1.110)<br>0.075	**0.295**<br>**(0.093,**<br>**0.933)**<br>**0.038**	**0.227**<br>**(0.061,**<br>**0.841)**<br>***0.026***	-	**0.279**<br>**(0.085,**<br>**0.910)**<br>***0.034***	**0.142**<br>**(0.027,**<br>**0.731)**<br>**0.020**
Gait speed (Clin)	OR<br>95%CI<br>*p*	**2.068**<br>**(1.282,**<br>**3.337)**<br>**0.003**	**1.448**<br>**(1.025,**<br>**2.045)**<br>**0.036**	-	**1.678**<br>**(1.137,**<br>**2.477)**<br>***0.009***	**1.867**<br>**(1.224,**<br>**2.847)**<br>***0.004***	**1.682**<br>**(1.107,**<br>**2.555)**<br>***0.015***
Knee alignment (Clin)	OR<br>95%CI<br>*p*	1.184<br>(0.990,<br>1.416)<br>*0.065*	**1.224**<br>**(1.015,**<br>**1.475)**<br>**0.034**	1.208<br>(0.994,<br>1.469)<br>*0.057*	-	**1.212**<br>**(1.005,**<br>**1.461)**<br>***0.044***	**1.382**<br>**(1.076,**<br>**1.775)**<br>***0.011***
Ankle/subtalar joint eversion (Clin)	OR<br>95%CI<br>*p*	-	-	-	-	-	-
Foot progression angle (Clin)	OR<br>95%CI<br>*p*	-	-	-	-	-	-

Gait speed (Lab)	OR<br>95%CI<br>*p*	**2.200**<br>**(1.332, 3.633)**<br>***0.002***	**1.488**<br>**(1.043, 2.121)**<br>***0.028***	-	**1.775**<br>**(1.182, 2.665)**<br>***0.006***	**1.978**<br>**(1.277, 3.065)**<br>***0.002***	**1.815**<br>**(1.162, 2.835)**<br>***0.009***
Knee alignment (Lab)	OR<br>95%CI<br>*p*	-	1.109<br>(0.984, 1.249)<br>*0.091*	-	-	-	**1.277**<br>**(1.072, 1.522)**<br>***0.006***
Ankle/subtalar joint eversion (Lab)	OR<br>95%CI<br>*p*	-	-	-	-	-	-
Foot progression angle (Lab)	OR<br>95%CI<br>*p*	-	-	-	-	-	-

Abbreviations: BMI = body mass index, FFI = foot function index, FPI = foot posture index, KL = Kellgren & Lawrence, OR = odds ratio
