# Supplementary material for: Clinically-accessible and laboratory-derived predictors of biomechanical response to standalone and supported lateral wedge insoles in people with knee osteoarthritis
Source: J Foot Ankle Res. 2023 Oct 26;16:74. doi: 10.1186/s13047-023-00671-7 (PMC10601168; doi:10.1186/s13047-023-00671-7)
Supplement: Supplementary file 4 — Additional file 4: Supplementary File 4a. AIC-selected logistic regression model statistics for 6% response threshold. Supplementary File 4b. AIC-selected logistic regression model statistics for 10% response threshold. [file 13047_2023_671_MOESM4_ESM.pdf]

Supplementary File 4a. AIC-selected logistic regression model statistics for 6% response threshold. Bolded odds ratio values and p-values indicate statistical significance (α = 0.05).

		WEDG		WEDG+V-ARCH	
		**Clinically-Accessible**	**Laboratory Derived**	**Clinically-Accessible**	**Laboratory Derived**
Responder : Non-Responder		22:31	22:31	20:33	20:33
Model AIC		68.786	68.998	58.340	57.563
Model Likelihood Ratio (p-value)	*p*	***0.010***	***0.011***	***<0.001***	***<0.001***
H&L Goodness of Fit (p-value)	*p*	0.622	0.195	0.180	0.127
AUC ROC (c)	c	0.736	0.717	0.864	0.861
	95%CI	(0.600, 0.875)	(0.575, 0.862)	(0.749, 0.978)	(0.747, 0.974)
**Odds Ratios by Predictor Variable**					
		Knee Align (Clin)	**Gait Speed (Lab)**	**Gait Speed (Clin)**	**Gait Speed (Lab)**
	OR	1.203	**1.438**	**1.800**	**2.003**
	95%CI	(0.995, 1.455)	**(1.005, 2.056)**	**(1.162, 2.787)**	**(1.247, 3.217)**
	p	*0.056*	***0.047***	***0.008***	***0.004***
		Gait Speed (Clin)	KL Grade	KL Grade	**Sex**
	OR	1.400	0.329	0.330	**0.118**
	95%CI	(0.985, 1.990)	(0.099, 1.094)	(0.073, 1.499)	**(0.017, 0.794)**
	p	*0.061*	*0.070*	*0.151*	***0.028***
				**Sex**	**KL Grade**
	OR			**0.149**	**0.201**
	95%CI			**(0.026, 0.866)**	**(0.044, 0.928)**
	p			***0.034***	***0.040***
				Knee Align (Clin)	Ankle Eversion (Lab)
	OR			1.221	1.274
	95%CI			(0.965, 1.545)	(0.936, 1.736)
	p			*0.097*	*0.124*

Abbreviations: AIC = Akaike information criterion, AUC = Area under curve, BMI = body mass index, FFI = Foot function index, FPI = foot posture index, H&L = Hosmer & Lemeshow, KL = Kellgren & Lawrence, OR = odds ratio, ROC = Receiver operating characteristic

Supplementary File 4b. AIC-selected logistic regression model statistics for 10% response threshold. Bolded odds ratio values and p-values indicate statistical significance (α = 0.05).

		WEDG		WEDG+V-ARCH	
		**Clinically-Accessible**	**Laboratory Derived**	**Clinically-Accessible**	**Laboratory Derived**
Responder : Non-Responder		16:37	16:37	12:41	12:41
Model AIC		61.298	63.249	37.917	30.071
Model Likelihood Ratio (p-value)	*p*	***0.009***	***0.022***	***<0.001***	***<0.001***
H&L Goodness of Fit (p-value)	*p*	0.846	0.670	0.354	0.999
AUC ROC (c)	c	0.782	0.731	0.968	0.976
	95%CI	(0.642, 0.922)	(0.575, 0.887)	(0.920, 1.000)	(0.937, 1.000)
**Odds Ratios by Predictor Variable**					
		KL Grade	**KL Grade**	**Knee Align (Clin)**	**Knee Align (Lab)**
	OR	0.250	**0.183**	**2.308**	**2.890**
	95%CI	(0.060, 1.045)	**(0.046, 0.734)**	**(1.182, 4.666)**	**(1.069, 7.810)**
	p	*0.058*	***0.016***	***0.015***	***0.036***
		Age	Age	**Gait Speed (Clin)**	**Gait Speed (Lab)**
	OR	1.109	1.072	**2.502**	**3.944**
	95%CI	(0.995, 1.237)	(0.973, 1.181)	**(1.177, 5.316)**	**(1.200, 12.965)**
	p	*0.061*	*0.157*	***0.017***	***0.024***
		Knee Align (Clin)		**Age**	Sex
	OR	1.253		**1.264**	0.001
	95%CI	(0.986,1.591)		**(1.012, 1.577)**	(<0.001, 1.071)
	p	*0.065*		***0.038***	*0.052*
				KL Grade	KL Grade
	OR			0.078	0.023
	95%CI			(0.005, 1.147)	(<0.001, 1.159)
	p			*0.063*	*0.059*
				Sex	Age
	OR			0.113	1.222
	95%CI			(0.009, 1.440)	(0.985, 1.517)
	p			*0.093*	*0.068*

Abbreviations: AIC = Akaike information criterion, AUC = Area under curve, BMI = body mass index, FFI = Foot function index, FPI = foot posture index, H&L = Hosmer & Lemeshow, KL = Kellgren & Lawrence, OR = odds ratio, ROC = Receiver operating characteristic
