# Supplementary material for: Clinically-accessible and laboratory-derived predictors of biomechanical response to standalone and supported lateral wedge insoles in people with knee osteoarthritis
Source: J Foot Ankle Res. 2023 Oct 26;16:74. doi: 10.1186/s13047-023-00671-7 (PMC10601168; doi:10.1186/s13047-023-00671-7)
Supplement: Supplementary file 5 — Additional file 5: Supplementary File 5. Overview of findings using force-entry modelling. [file 13047_2023_671_MOESM5_ESM.pdf]

**Supplementary File 5. Overview of findings using force-entry modelling.**

The logistic regression models for predicting a 2% KAM impulse reduction with WEDG and WEDG+V-ARCH insole conditions were significant ($p < 0.05$) using forced-entry of clinically-accessible and laboratory-derived pools of predictor variables. Model statistics and odds ratios for predictor variables at the 2% response threshold are summarized in below in Supplementary File 5a. At the 6% (Supplementary File 5b) and 10% (Supplementary File 5c) response thresholds, no models were significant for WEDG ($p > 0.134$), and only the laboratory-derived model at 6% was significant for WEDG+V-ARCH. Both models were significant for WEDG+V-ARCH at 10%, though sex was excluded from the final model due to a lack of model convergence.

For the WEDG condition at the 2% response threshold, clinically-accessible and laboratory-derived gait speed was a significant predictor such that a faster gait speed was related to a greater likelihood of reducing the KAM impulse. NRS pain was the only other significant predictor in the clinically-accessible model for WEDG, such that higher knee pain was related to a greater likelihood of KAM impulse reduction. Predictor variables of interest ($p < 0.10$) potentially linked to a greater likelihood of KAM impulse reduction included older age for the clinically-accessible model, and higher NRS pain and lower FFI pain for the laboratory-derived model.

For the WEDG+V-ARCH condition at the 2% response threshold, significant predictor variables for clinically-accessible and laboratory-derived models included gait speed and sex. Having faster gait speed and female sex were related to an increased likelihood of reducing the KAM impulse with WEDG+V-ARCH. NRS pain was also a significant predictor for the clinically-accessible model, such that higher knee pain was related to greater likelihood of KAM

impulse reduction with this insole. Predictor variables of interest ($p < 0.10$) potentially linked to a greater likelihood of KAM impulse reduction included lower FFI pain for both clinically-accessible and laboratory-derived models, and increased NRS pain for the laboratory-derived model only.

In other significant models at 6% and 10% KAM reduction thresholds, faster gait speeds and female sex were generally seen to be predictive of KAM response when wearing WEDG+V-ARCH.

**Supplementary File 5a. Forced-entry logistic regression model statistics for 2% response threshold.** Bolded odds ratio values and p-values indicate statistical significance (α = 0.05).

| | | WEDG | | WEDG+V-ARCH | |
|---|---|---|---|---|---|
| | | **Clinically-Accessible** | **Laboratory Derived** | **Clinically-Accessible** | **Laboratory Derived** |
| Responder : Non-Responder | | 33:20 | 33:20 | 28:25 | 28:25 |
| Model AIC | | 65.652 | 68.375 | 73.601 | 75.379 |
| Model Likelihood Ratio (p-value) | *p* | ***0.003*** | ***0.007*** | ***0.014*** | ***0.025*** |
| H&L Goodness of Fit (p-value) | *p* | *0.670* | *0.550* | *0.199* | *0.571* |
| AUC ROC (c) | c | 0.874 | 0.880 | 0.861 | 0.847 |
| | 95%CI | (0.780, 0.968) | (0.789, 0.972) | (0.762, 0.961) | (0.738, 0.957) |
| | | **Odds Ratios by Predictor Variable** | | | |
| Age | OR | 1.141 | 1.072 | 1.074 | 1.048 |
| | 95%CI | (0.978, 1.331) | (0.936, 1.226) | (0.945, 1.222) | (0.934, 1.176) |
| | *p* | *0.093* | *0.316* | *0.275* | *0.429* |
| Sex | OR | 1.041 | 0.716 | **0.148** | **0.121** |
| | 95%CI | (0.158, 6.858) | (0.091, 5.656) | **(0.023, 0.961)** | **(0.015, 0.970)** |
| | *p* | *0.966* | *0.751* | ***0.045*** | ***0.047*** |
| BMI | OR | 0.958 | 0.960 | 0.985 | 1.004 |
| | 95%CI | (0.755, 1.214) | (0.763, 1.207) | (0.792, 1.225) | (0.809, 1.247) |
| | *p* | *0.721* | *0.724* | *0.892* | *0.968* |
| FPI | OR | 0.948 | 0.896 | 0.995 | 0.941 |
| | 95%CI | (0.751, 1.196) | (0.720, 1.116) | (0.807, 1.227) | (0.772, 1.148) |
| | *p* | *0.652* | *0.328* | *0.964* | *0.551* |
| NRS pain | OR | **2.256** | 1.692 | **1.984** | 1.538 |
| | 95%CI | **(1.041, 4.891)** | (0.943, 3.036) | **(1.070, 3.681)** | (0.939, 2.520) |
| | *p* | ***0.039*** | *0.078* | ***0.030*** | *0.087* |
| FFI pain | OR | 0.950 | 0.949 | 0.950 | 0.955 |
| | 95%CI | (0.890, 1.013) | (0.896, 1.006) | (0.899, 1.004) | (0.906, 1.008) |
| | *p* | *0.118* | *0.080* | *0.069* | *0.093* |
| KL grade | OR | 0.423 | 0.319 | 0.812 | 0.576 |
| | 95%CI | (0.073, 2.462) | (0.052, 1.961) | (0.170, 3.870) | (0.124, 2.667) |
| | *p* | *0.339* | *0.218* | *0.794* | *0.480* |
| Gait speed | OR | **4.060** | **3.001** | **2.710** | **2.288** |
| | 95%CI | **(1.551, 10.628)** | **(1.466, 6.144)** | **(1.329, 5.524)** | **(1.271, 4.117)** |
| | *p* | ***0.004*** | **0.003** | **0.006** | **0.006** |
| Knee alignment | OR | 1.279 | 1.035 | 1.190 | 1.032 |
| | 95%CI | (0.939, 1.742) | (0.846, 1.267) | (0.904, 1.566) | (0.873, 1.220) |
| | *p* | *0.118* | *0.737* | *0.215* | *0.711* |

| | | | | | |
|---|---|---|---|---|---|
| Ankle/subtalar joint eversion | OR | 0.899 | 1.018 | 0.876 | 1.101 |
| | 95%CI | (0.703, 1.150) | (0.735, 1.410) | (0.704, 1.090) | (0.818, 1.481) |
| | *p* | *0.397* | *0.913* | *0.234* | *0.526* |
| Foot progression angle | OR | 0.875 | 0.922 | 0.909 | 1.006 |
| | 95%CI | (0.732, 1.047) | (0.802, 1.059) | (0.779, 1.060) | (0.888, 1.139) |
| | *p* | *0.145* | *0.250* | *0.223* | *0.924* |

Abbreviations: AIC = Akaike information criterion, AUC = area under curve, BMI = body mass index, FFI = foot function index, FPI = foot posture index, H&L = Hosmer & Lemeshow, KL = Kellgren & Lawrence, OR = odds ratio, ROC = receiver operating characteristic.

**Supplementary File 5b. Forced-entry logistic regression model statistics for 6% response threshold.** Bolded odds ratio values and p-values indicate statistical significance (α = 0.05). Only significant models (p < 0.05) with appropriate goodness of fit (p > 0.05) are included.

| | | WEDG | | WEDG+V-ARCH | |
|---|---|---|---|---|---|
| | | **Clinically-Accessible** | **Laboratory Derived** | **Clinically-Accessible** | **Laboratory Derived** |
| Responder : Non-Responder | | 22:31 | 22:31 | 20:33 | 20:33 |
| Model AIC | | 79.748 | 81.448 | 69.435 | 68.894 |
| Model Likelihood Ratio (p-value) | *p* | *0.134* | *0.207* | ***0.010*** | ***0.008*** |
| H&L Goodness of Fit (p-value) | *p* | *0.668* | *0.558* | ***0.013*** | *0.608* |
| AUC ROC (c) | c | 0.804 | 0.774 | 0.882 | 0.880 |
| | 95%CI | (0.687, 0.920) | (0.649, 0.900) | (0.776, 0.988) | (0.780, 0.980) |
| | | **Odds Ratios by Predictor Variable** | | | |
| Age | OR | - | - | - | 1.052 |
| | 95%CI | | | | (0.921, 1.200) |
| | *p* | | | | *0.456* |
| Sex | OR | - | - | - | **0.072** |
| | 95%CI | | | | **(0.007, 0.736)** |
| | *p* | | | | ***0.027*** |
| BMI | OR | - | - | - | 0.973 |
| | 95%CI | | | | (0.767, 1.234) |
| | *p* | | | | *0.821* |
| FPI | OR | - | - | - | 1.040 |
| | 95%CI | | | | (0.842, 1.284) |
| | *p* | | | | *0.716* |
| NRS pain | OR | - | - | - | 1.229 |
| | 95%CI | | | | (0.733, 2.062) |
| | *p* | | | | *0.434* |
| FFI pain | OR | - | - | - | 1.010 |
| | 95%CI | | | | (0.951, 1.071) |
| | *p* | | | | *0.753* |
| KL grade | OR | - | - | - | 0.230 |
| | 95%CI | | | | (0.040, 1.312) |
| | *p* | | | | *0.098* |
| Gait speed | OR | - | - | - | **2.146** |
| | 95%CI | | | | **(1.210, 3.805)** |
| | *p* | | | | ***0.009*** |
| Knee alignment | OR | - | - | - | 1.102 |
| | 95%CI | | | | (0.915, 1.328) |

| | | | | | |
|---|---|---|---|---|---|
| | *p* | | | | *0.306* |
| Ankle/subtalar joint eversion | OR | | | | 1.246 |
| | 95%CI | - | - | - | (0.885, 1.754) |
| | *p* | | | | *0.208* |
| Foot progression angle | OR | | | | 1.037 |
| | 95%CI | - | - | - | (0.914, 1.176) |
| | *p* | | | | *0.575* |

Abbreviations: AIC = Akaike information criterion, AUC = area under curve, BMI = body mass index, FFI = foot function index, FPI = foot posture index, H&L = Hosmer & Lemeshow, KL = Kellgren & Lawrence, OR = odds ratio, ROC = receiver operating characteristic

**Supplementary File 5c. Forced-entry logistic regression model statistics for 10% response threshold.** Bolded odds ratio values and p-values indicate statistical significance (α = 0.05). Only significant models (p < 0.05) with appropriate goodness of fit (p > 0.05) are included.

| | | WEDG | | WEDG+V-ARCH | |
|---|---|---|---|---|---|
| | | Clinically-Accessible | Laboratory Derived | Clinically-Accessible | Laboratory Derived * |
| Responder : Non-Responder | | 16:37 | 16:37 | 12:41 | 12:41 |
| Model AIC | | 75.340 | 77.550 | 45.780 | 47.011 |
| Model Likelihood Ratio (p-value) | *p* | *0.257* | *0.413* | ***<0.001*** | ***<0.001*** |
| H&L Goodness of Fit (p-value) | *p* | *0.923* | *0.678* | *0.822* | *0.388* |
| AUC ROC (c) | c | 0.812 | 0.770 | 0.968 | 0.949 |
| | 95%CI | (0.670, 0.927) | (0.631, 0.909) | (0.923, 1.000) | (0.895, 1.000) |
| **Odds Ratios by Predictor Variable** | | | | | |
| Age | OR | | | 1.217 | 1.141 |
| | 95%CI | - | - | (0.938, 1.578) | (0.934, 1.394) |
| | *p* | | | *0.140* | 0.197 |
| Sex | OR | | | 0.011 | |
| | 95%CI | - | - | (<0.001, 6.149) | - |
| | *p* | | | *0.162* | |
| BMI | OR | | | 0.658 | 0.838 |
| | 95%CI | - | - | (0.306, 1.417) | (0.581, 1.209) |
| | *p* | | | *0.286* | 0.345 |
| FPI | OR | | | 0.708 | 0.854 |
| | 95%CI | - | - | (0.385, 1.303) | (0.629, 1.159) |
| | *p* | | | *0.267* | 0.310 |
| NRS pain | OR | | | 0.778 | 0.774 |
| | 95%CI | - | - | (0.347, 1.742) | (0.366, 1.637) |
| | *p* | | | *0.541* | 0.503 |
| FFI pain | OR | | | 1.006 | 1.020 |
| | 95%CI | - | - | (0.856, 1.182) | (0.918, 1.133) |
| | *p* | | | *0.945* | 0.711 |
| KL grade | OR | | | 0.106 | 0.081 |
| | 95%CI | - | - | (0.002, 4.711) | (0.005, 1.421) |
| | *p* | | | *0.246* | 0.086 |
| Gait speed | OR | | | 1.797 | **2.286** |
| | 95%CI | - | - | (0.690, 4.679) | **(1.077, 4.853)** |
| | *p* | | | *0.230* | **0.031** |
| Knee alignment | OR | - | - | **2.388** | **1.479** |
| | 95%CI | | | **(1.041, 5.478)** | **(1.036, 2.112)** |

| | | | | | |
|---|---|---|---|---|---|
| | *p* | | | ***0.040*** | **0.031** |
| Ankle/subtalar joint eversion | OR | | | 1.274 | 1.037 |
| | 95%CI | - | - | (0.724, 2.242) | (0.620, 1.735) |
| | *p* | | | *0.401* | 0.890 |
| Foot progression angle | OR | | | 1.081 | 0.937 |
| | 95%CI | - | - | (0.838, 1.395) | (0.752, 1.167) |
| | *p* | | | *0.547* | 0.559 |

* Indicates the Laboratory-Derived model for WEDG+V-ARCH used a pool of forced-entry predictor variables that excluded sex. The model which included sex was not reported because it did not converge statistically.

Abbreviations: AIC = Akaike information criterion, AUC = area under curve, BMI = body mass index, FFI = foot function index, FPI = foot posture index, H&L = Hosmer & Lemeshow, KL = Kellgren & Lawrence, OR = odds ratio, ROC = receiver operating characteristic
